# The effect of self-directed learning ability on core competence in undergraduate nursing students: the mediating role of professional self-concept

**DOI:** 10.3389/fpsyg.2026.1892419

**Published:** 2026-07-22

**Authors:** Lulu Zheng, Li Li, Yuantong Zang, Weiyue Cong, Na Li, Guanzhong Yan

**Affiliations:** 1 School of Nursing, Inner Mongolia Medical University, Hohhot, China; 2 Second Affiliated Hospital of Inner Mongolia Medical University, Hohhot, China

**Keywords:** core competence, professional self-concept, self-directed learning, mediation analysis, nursing education

## Abstract

**Background:**

Core competence is a key indicator of nursing students' clinical readiness. While self-directed learning and professional self-concept are correlated with this competence, their underlying mechanisms remain unclear.

**Purpose:**

To examine the association between self-directed learning and undergraduate nursing students' core competence and the mediating role of professional self-concept in this relationship.

**Methods:**

A cross-sectional survey of 237 undergraduate nursing students from a medical university in northern China was conducted. Data collected via structured scales were analyzed using correlation, path analysis, and bootstrapping.

**Results:**

Self-directed learning, professional self-concept, and core competence were all at moderate levels and significantly positively correlated (*P* < 0.001). Professional self-concept partially mediated the effect of self-directed learning on core competence (indirect effect = 0.422), accounting for 24.4% of the total effect.

**Conclusions:**

Self-directed learning is positively related to core competence both directly and indirectly through professional self-concept as a partial mediator. Educators should consider fostering active learning and professional identity to better prepare students for clinical practice.

## 1 Introduction

Cultivating a high-quality nursing workforce is one of the key measures to achieve the goals of optimizing health services and building a healthy environment in the Healthy China 2030 Blueprint (Tang et al., 2021). It is also a common challenge for nursing educators worldwide, and workforce development should prioritize the cultivation of core competence (Liu, 2017). Core competence in undergraduate nursing students integrates knowledge, skills, attitudes, values, and personal traits (Zhou and Zeng, 2017). Nursing students are required to possess core qualities including interpersonal relationships, decision-making ability, ethical values, critical thinking, and problem-solving skills (Çalişkan et al., 2025). As a primmary objective of nursing education, it significantly associated with students' future professional performance (Tseng et al., 2022). Studies have shown that nursing colleges should attach great importance to the cultivation of nursing students' core competencies, and undergraduate teaching reform should highlight the advantages of undergraduate nursing education to systematically improve nursing students' core competencies (Zhou et al., 2025). This emphasis aligns with the current international consensus on nursing education, which calls for strengthening the systematic training of core competencies to ensure that graduates can adapt to evolving healthcare needs (Wang et al., 2026), and is reflected in the broader movement toward competency-based frameworks adopted across Europe and other regions in recent years (Mani, 2025). However, current evidence indicates that nursing undergraduates generally exhibit only a moderate level of core competence, with notable deficiencies in critical thinking and research capabilities (Ma et al., 2015). These gaps hinder their transition into competent clinical practitioners and may lead to inadequate preparedness of newly recruited nurses in clinical decision-making, teamwork, and patient safety.

Self-directed learning ability allows nursing students-driven by intrinsic motivation and positive attitudes—to actively seek and utilize resources to master requisite professional knowledge and skills (Siqueira et al., 2020). Evidence indicates that undergraduates with strong self-directed learning skills often perform better in their subsequent nursing careers (Du et al., 2023). A recent multi-country study has also identified self-directed learning readiness as a significant correlate of self-assessed competence among graduating nursing students across Europe (Visiers-Jiménez et al., 2022). However, the specific mechanisms underlying the relationship between self-directed learning and sustainable core competence, and whether an essential psychological transitional pathway mediates this relationship, warrant deeper exploration. Professional self-concept refers to students' evolving perceptions of the nursing profession as they transition from undergraduates to registered nurses. As a foundational professional attitude, it encompasses an individual's cognitive understanding, self-esteem, and behavioral orientation toward nursing (Arthur, 1995). A positive professional self-concept is associated with students' career commitment and their resilience against negative public perceptions (Li et al., 2013).

Bandura's Triadic Reciprocal Determinism theory posits a dynamic and continuous interaction among personal factors (cognition, motivation, and emotion), behavior, and the environment (Bandura, 1986). Grounded in this framework, our study conceptualizes self-directed learning as the cognitive and behavioral foundation for nursing undergraduates, professional self-concept as their professional cognition and emotion, and core competence as the ultimate behavioral outcome. In this conceptualization, professional self-concept is positioned as the personal cognitive factor that translates active learning behaviors into professional competence, making it a conceptually appropriate mediator. However, a cross-sectional design cannot statistically rule out alternative directional paths. Alternative model specifications—such as reversing the direction of mediation—would be less consistent with the theoretical and temporal ordering of these constructs, as self-directed learning represents a foundational capacity that develops early, whereas professional self-concept emerges through ongoing learning and socialization. Consequently, a strong intrinsic correlation among these three constructs is hypothesized. Although one study suggests positive correlations between professional self-concept, self-directed learning, and core competence (Ge et al., 2024), this study predominantly focused on nursing interns—defined as fourth-year nursing students undertaking full-time clinical rotations in hospitals—rather than pre-internship undergraduates. Furthermore, the specific mediating role of professional self-concept in linking self-directed learning to core competence remains largely unexplored.

To better understand the internal mechanisms underlying core competence in nursing undergraduates and to inform more targeted educational interventions, this study constructed and validated a mediation model grounded in Bandura's Triadic Reciprocal Determinism. The specific objectives were to: (1) assess the current levels of self-directed learning, professional self-concept, and core competence among nursing undergraduates; (2) analyze the correlations among these three constructs; and (3) examine the mediating role of professional self-concept between self-directed learning and core competence. Our findings aim to contribute to the understanding of nursing students' core competence from a mechanistic perspective, providing theoretical foundations and practical guidance for optimizing nursing talent cultivation models.

## 2 Methods

### 2.1 Study participants

A cross-sectional survey utilizing convenience sampling was conducted between March and April 2025 among undergraduate nursing students at a medical university in northern China. The program follows a 4-year curriculum, with the first 3 years dedicated to on-campus theoretical and laboratory-based training, and the fourth year devoted to full-time clinical internship. At the time of data collection, participants were second- or third-year students who had completed foundational coursework but had not yet entered clinical practice. The inclusion criteria were: (1) full-time undergraduate nursing students who had completed the first-year courses, were enrolled in the second or third-year courses, and had not yet started clinical practice during the survey period (March to April 2025). Exclusion criteria included: (2) students undergoing clinical internships, as the clinical practice environment has been shown to exert a significant impact on nursing students' professional self-concept (Chen et al., 2025).

According to the rule of thumb for sample size estimation, the minimum required sample should be five to 10 times the number of independent variables (Thomas, 2023). In this study, core competence served as the dependent variable, while 13 independent variables were identified (four dimensions of self-directed learning, five dimensions of professional self-concept, and four demographic characteristics: age, sex, only-child status, and class cadre status). Accounting for a 20% potential attrition rate, the target sample size was estimated to be between 82 and 163. However, given that SEM literature generally recommends a sample size of at least 200 for stable parameter estimation (Kline, 2023), the final sample of 237 valid responses was included. This study was approved by the Institutional Review Board (IRB) of the Second Affiliated Hospital of Inner Mongolia Medical University (Approval No. EFY20260049(3)). All participants were informed about the study's purpose, procedures, and confidentiality measures through a detailed information sheet on the first page of the online questionnaire. Participants provided informed consent by clicking the “Agree” option before proceeding, and were clearly informed that participation was strictly voluntary, and that they could withdraw at any time without any negative consequences. All data were collected anonymously, and the study was conducted in accordance with the Declaration of Helsinki.

### 2.2 Measures

#### 2.2.1 General information questionnaire

A researcher-designed demographic questionnaire was used to collect data on participants' age, sex, class year, only-child status, and whether they served as class cadres (Students who assist teachers in managing class affairs, such as class monitors and class representatives). This study included the only-child variable, given its potential association with students' psychological and behavioral characteristics, such as independence, a sense of responsibility, and learning autonomy. Existing studies have regarded it as an important demographic variable in nursing education (Luo et al., 2022).

#### 2.2.2 Self-directed learning ability scale for nursing students

Developed by Zhang (2007), this scale evaluates the self-directed learning capacity of nursing undergraduates. It comprises 30 items across four dimensions: learning motivation, self-management ability, cooperative ability, and information literacy. Items are rated on a 5-point Likert scale ranging from one (completely disagree) to five (completely agree). Items 10, 16, 20, 24, and 28 are reverse-scored. The scores range from 8–40 for learning motivation, 11–55 for self-management ability, 5–25 for cooperative ability, and 6–30 for information literacy. Total scores range from 30 to 150, with higher scores indicating stronger self-directed learning abilities. In this study, Cronbach's α coefficient was used to test the internal consistency reliability of the scale. The results showed that the Cronbach's α coefficient of the total scale was 0.886, indicating that the scale had good reliability in the sample of this study.

#### 2.2.3 Professional self-concept of nurses instrument (PSCNI)

Originally developed by Arthur (Arthur, 1992) in 1992, the PSCNI assesses nursing professionals' cognitive understanding, self-esteem, and behavioral orientation toward their profession. The scale comprises 30 items across five dimensions: leadership, skill, flexibility, satisfaction, and communication. Items are rated on a 4-point Likert scale from one (disagree) to four (agree), with items 9, 12, 13, 18, 21, 23, and 25 being reverse-scored. The score ranges are 4–16 for leadership, 5–20 for skills, 7–28 for flexibility, 9-36 for satisfaction, and 5–20 for communication. Total scores range from 30 to 120. A total score exceeding 75 (item mean > 2.50) indicates a positive professional attitude. The Chinese version of this scale was tested for reliability and validity by Yang et al. (2008) in 2008, with a split-half reliability of 0.86 and an internal consistency reliability of 0.84. It has good applicability among Chinese nursing students. The Chinese version of the scale adopted in this study is consistent with that used by Pu (2009). In this study, Cronbach's α coefficient was adopted to examine the internal consistency reliability of the scale. The results showed that the Cronbach's α coefficient was 0.914, indicating that the scale had good reliability among the research samples.

#### 2.2.4 Core competence scale for nursing students

Developed by Yan (2023) in 2022, this 39-item scale evaluates the core competence of nursing students across six dimensions: person-centered care, interdisciplinary teamwork, evidence-based practice, quality improvement, informatics, and professional values/traits. Items are rated on a 5-point Likert scale ranging from one (completely agree) to five (completely disagree). The score ranges are 13–65 for person-centered care, 4–20 for interdisciplinary teamwork, 6–30 for evidence-based practice, 4–20 for quality improvement, 3–15 for informatics, and 9–45 for values and traits. The total score ranges from 39 to 195. Originally, lower scores indicated higher competence. To facilitate interpretation and ensure directional consistency with the other variables, all items were reverse-scored prior to data analysis, such that higher converted scores reflect stronger core competence. In this study, Cronbach's α coefficient was used to assess the internal consistency reliability of the scale. The results revealed that the Cronbach's α coefficient of the scale was 0.978, indicating excellent reliability of the scale in the present study sample.

### 2.3 Data collection

An online survey was conducted using the Wenjuanxing platform. Participation was strictly voluntary, and the study's purpose and instructions were explicitly outlined on the first page of the questionnaire. Investigators distributed the survey link to the WeChat groups of nursing students across eight classes. To ensure data integrity, all items were set as mandatory, and responses were restricted to one per IP address. Upon completion of the survey, three researchers independently screened the collected data. Questionnaires exhibiting obvious patterned responses, identical selections across all items, or completion times of less than 100 s were excluded.

### 2.4 Statistical analysis

Data analysis was conducted using SPSS version 27.0 (IBM Corp). Continuous variables were described using means and standard deviations (*M* ± *SD*), while categorical variables were presented as frequencies and percentages. Pearson correlation analysis was employed to examine the relationships among self-directed learning ability, professional self-concept, and core competence. Structural equation modeling (SEM) was constructed using AMOS version 26.0 (IBM Corp) to analyze the mediation effects. In terms of model specification, all three core constructs were represented as latent variables with their respective dimension total scores as manifest indicators—four for self-directed learning, five for professional self-concept, and six for core competence. This approach maintains an acceptable indicator-to-sample-size ratio (15 indicators, *N* = 237) while preserving the multidimensional structure of each construct.

Prior to SEM analysis, preliminary assumption testing was conducted: univariate normality was assessed via skewness and kurtosis (all |skewness| < 2 and |kurtosis| < 7) (Byrne, 2016), multicollinearity was examined via the variance inflation factor (all VIF < 5) (Hair et al., 2010), and no influential outliers were identified based on Cook's distance (all < 1) (Cook and Weisberg, 1982) and standardized residuals (all within ± 3). These results confirmed that the assumptions for SEM were adequately met.

The significance of the mediation effects and their 95% confidence intervals (CIs) were estimated using the bias-corrected bootstrapping method with 5,000 resamples. A 2-sided *P* value of < 0.05 was considered statistically significant. Given that all data were collected through students' self-reports, common method bias might exist. Harman's single-factor test was therefore adopted in this study for bias diagnosis. All items from the three scales of self-directed learning ability, professional self-concept, and core competence were included in the unrotated principal component factor analysis. The results showed a total of 19 factors with eigenvalues >1 were extracted, and the first factor explained 34.083% of the total variance, which was lower than the critical value of 40%. Harman's single-factor test was conducted as a supplementary diagnostic (Harman, 1967). However, given the limitations of this test and the cross-sectional self-report design, residual common method bias cannot be completely ruled out.

## 3 Results

### 3.1 Demographic characteristics

Of the 248 distributed questionnaires, 237 valid responses were retained, yielding an effective response rate of 95.56%. A total of 237 nursing undergraduates were included in this study. Their ages ranged from 20 to 25 years, with a mean (*SD*) age of 21.59 (1.06) years. The sample comprised 40 male students (16.9%) and 197 female students (83.1%). Regarding family background, 79 participants (33.3%) were only children, and 158 (66.7%) were not. Additionally, 47 students (19.8%) served as class cadres, whereas 190 (80.2%) did not.

### 3.2 Descriptive statistics and correlation analysis

As shown in Table 1, the mean (*SD*) total score for self-directed learning ability was 107.17 (12.15), total score range: 30–150, with an item mean of 3.57, indicating an above-average level. Among its dimensions, information literacy scored the highest (item mean, 3.61), while cooperative ability scored the lowest (item mean, 3.46). The mean (*SD*) total score for professional self-concept was 84.24 (10.82); score range: 30–120, with an item mean of 2.81, reflecting a moderate level. Across its five dimensions, flexibility (item mean, 3.03) and skill (item mean, 3.02) yielded relatively higher scores, whereas satisfaction recorded the lowest (item mean, 2.54). After reverse-scoring conversion, the mean (*SD*) total score for core competence was 143.72 (23.38), score range: 39–195, with an item mean of 3.69, suggesting an above-average level. The professional values and traits dimension achieved the highest score (item mean, 3.87), whereas evidence-based practice scored the lowest (item mean, 3.46; Table 1).

**Table 1:** Descriptive statistics and correlations among total scores of key variables (*N* = 237).

Variable	Possible range	Mean (*SD*)	Item mean	1	2
1. Self-directed learning ability	30–50	107.17(12.15)	3.57	–	
2. Professional self-concept	30–120	84.24 (10.82)	2.81	0.612^***^	–
3. Core competence	39–195	143.72(23.38)	3.69	0.692^***^	0.634^***^

^*^Item mean was calculated by dividing the total score by the number of items. ^***^P < 0.001.

Pearson correlation analysis revealed significant positive correlations among self-directed learning ability, professional self-concept, and core competence (all *P* < 0.001). Specifically, self-directed learning ability demonstrated the strongest correlation with core competence (*r* = 0.692). Professional self-concept was also strongly correlated with core competence (*r* = 0.634), and a moderate positive correlation was observed between self-directed learning ability and professional self-concept (*r* = 0.612; Table 1).

### 3.3 Mediation analysis

**Figure 1:**
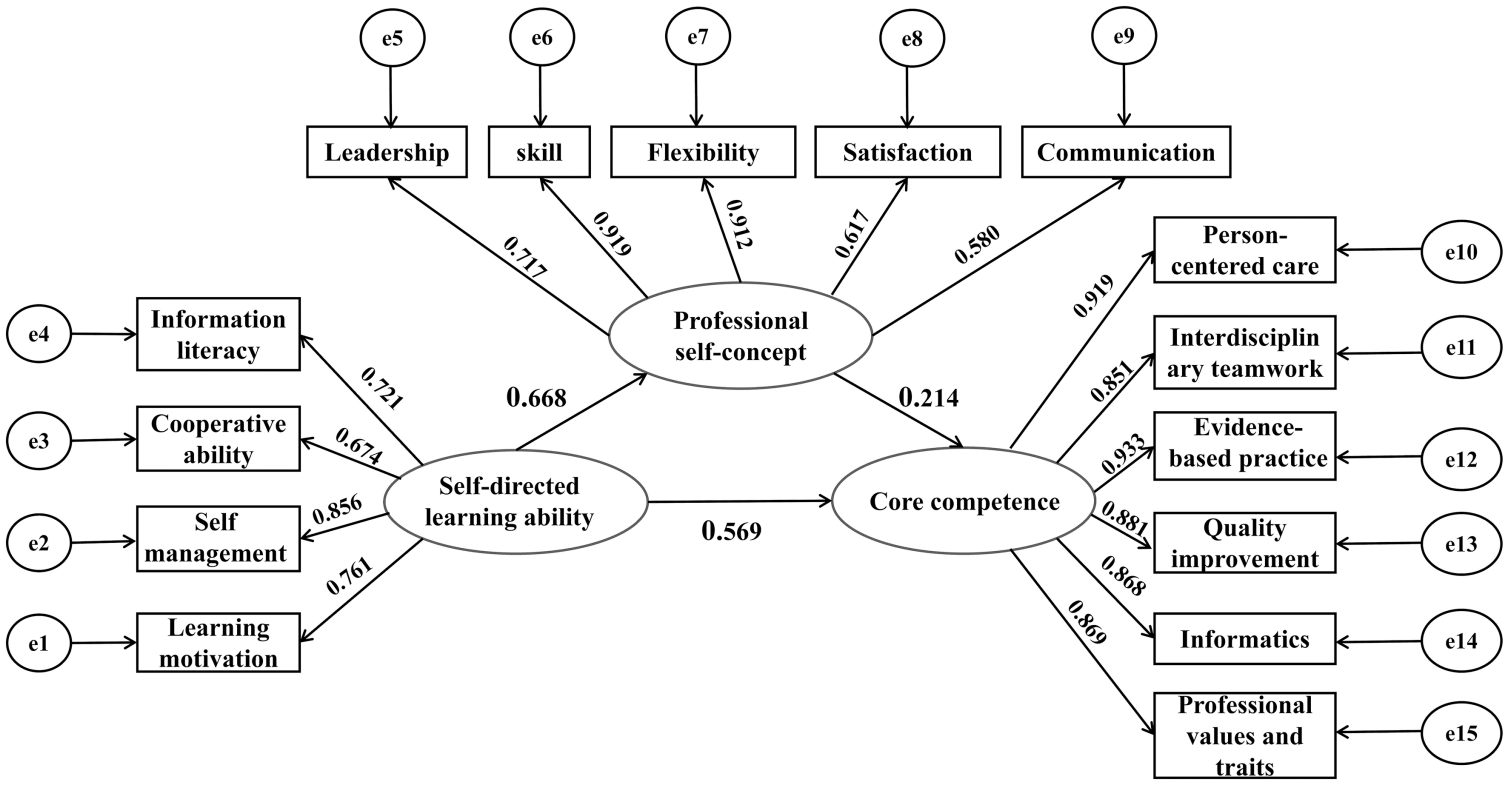
Mediation effect model of professional self-concept between self-directed learning ability and core competence.

Structural equation modeling (SEM) was performed using AMOS 26.0 to establish a path model, with self-directed learning ability as the independent variable, core competence as the dependent variable, and professional self-concept as the mediator. Based on theoretical considerations and supported by modification indices, covariances were added between two sets of residual errors: (a) e10 and e12, corresponding to person-centered care and evidence-based practice—both patient-oriented clinical competencies that are complementary in modern nursing; and (b) e12 and e15, corresponding to evidence-based practice and professional values/traits—both reflecting the scientific and ethical foundations of nursing practice. These residual correlations were introduced as *post-hoc* model modifications guided by theoretical interpretability, with modification indices consulted only as secondary diagnostics. Only one minimal adjustment round was performed to avoid overfitting, and the modifications were justified by meaningful relationships between the corresponding dimensions rather than by statistical fit alone. The final model is illustrated in Figure 1. Model fit indices indicated an acceptable overall fit: the ratio of chi-square to degrees of freedom *(*χ^2^*/df* ) was 1.890 (< 3); root mean square error of approximation (RMSEA) = 0.061 (< 0.08); goodness-of-fit index (GFI) = 0.921 (>0.90); comparative fit index (CFI) = 0.974 (>0.90); and Tucker-Lewis index (TLI) = 0.967 (>0.90). Although the adjusted goodness-of-fit index (AGFI = 0.887) was slightly below the conventional threshold of 0.90, all other indices demonstrated good fit.

As shown in the path analysis (Figure 1), self-directed learning ability and professional self-concept were significantly positively associated (β = 0.668, *P* < 0.001). Professional self-concept also showed a significant positive association with core competence (β = 0.214, *P* < 0.001). After controlling for the mediator, the association between self-directed learning ability on core competence remained significant (β = 0.569, *P* < 0.001).

To further verify the significance of the mediation effect, a bias-corrected bootstrapping method with 5,000 resamples was applied. The unstandardized total effect was 1.733 (95% CI, 1.313–2.163; *P* < 0.001), and the direct effect was 1.311 (95% CI, 0.848–1.851; *P* < 0.001). The indirect effect was 0.422 (95% CI, 0.187–0.696; *P* = 0.002). The indirect effect accounted for 24.4% of the total effect (95% CI, 8.9%-40.7%; *P* = 0.003). As the 95% CI for the indirect effect did not include zero, professional self-concept demonstrated significant partial mediating pathway between self-directed learning ability and core competence (Table 2).

**Table 2:** Descriptive statistics for sub-dimensions of key variables (*N* = 237).

Variable/Sub-dimension	Possible range	Mean (SD)	Item mean
Self-directed learning ability			
Learning motivation	8–40	28.72 (4.26)	3.59
Self-management ability	11–55	39.49 (5.20)	3.59
Cooperative ability	5–25	17.30 (2.15)	3.46
Information literacy	6–30	21.65 (2.90)	3.61
Professional self-concept			
Leadership	4–16	11.19 (1.86)	2.80
Skill	5–20	15.11 (2.25)	3.02
Flexibility	7–28	21.21 (3.09)	3.03
Satisfaction	9–36	22.89 (4.39)	2.54
Communication	5–20	13.85 (1.78)	2.77
Core competence			
Person-centered care	13–65	48.15 (8.12)	3.70
Interdisciplinary teamwork	4–20	14.19 (2.48)	3.55
Evidence-based practice	6–30	20.77 (4.32)	3.46
Quality improvement	4–20	14.95 (2.66)	3.74
Informatics	3–15	10.82 (2.19)	3.61
Professional values and traits	9–45	34.84 (6.07)	3.87

The mean was calculated by dividing the total score by the number of items.

### 3.4 Measurement model evaluation

The measurement model demonstrated adequate psychometric properties. Standardized factor loadings ranged from 0.580 to 0.933 (all *P* < 0.001). Composite reliability (*CR*) values ranged from 0.849 to 0.956, and average variance extracted (*AVE*) values ranged from 0.586 to 0.787, exceeding the recommended thresholds for convergent validity (Fornell and Larcker, 1981). Additionally, the square roots of the AVE for each construct were greater than the corresponding inter-construct correlations, supporting adequate discriminant validity (Hair et al., 2019).

## 4 Discussion

### 4.1 Current status of key variables among nursing undergraduates

The results of this study indicated that the scores for core competence, self-directed learning ability, and professional self-concept among nursing undergraduates were generally at a moderate to above-average level. Core competence scored the highest, which provided some preliminary evidence suggesting that the nursing education program at the participating university shows a statistical association with students' clinical skills and professional values. The high scores in the dimensions of “person-centered care” and “professional values and traits” are consistent with the current emphasis on the cultivation of students' humanistic literacy in nursing education. Furthermore, this reflects that with the shift toward a “patient-centered” medical model, humanistic caring ability has become a core requirement of nursing professionalism (Feng et al., 2025). The above-average level of self-directed learning ability indicates that nursing students possess a certain degree of self-directed learning awareness and capacity. The highest score in the “information literacy” dimension is consistent with the emphasis on information-based teaching methods in nursing practical training (Li, 2019). Interestingly, this contrasts with a recent Nordic study reporting that graduating nursing students rated their eHealth competence lowest among all clinical competencies, suggesting that the development of information-related skills may vary considerably across educational and cultural contexts (Eronen et al., 2025).

However, the relatively lower score in “cooperative ability” suggests room for improvement in teamwork-based learning. Although professional self-concept was at a moderate level, the “satisfaction” dimension scored the lowest, indicating that nursing students in this study still need to improve their professional identity and career satisfaction with the nursing profession. Interestingly, these findings differ from a study by Ge et al. (2024) on nursing interns, which reported a more positive professional self-concept but lower self-directed learning and core competence. This discrepancy may be related to fact that on-campus nursing students have not yet experienced the role transition and reality shock of clinical practice; thus, their self-evaluations are largely based on theoretical learning and classroom performance. This highlights the need for educators to strengthen clinical adaptability and reflective learning guidance prior to internships, facilitating a smooth transition from theory to practice.

### 4.2 Correlations among self-directed learning ability, professional self-concept, and core competence

Correlation analysis demonstrated significant positive associations among self-directed learning ability, professional self-concept, and core competence. Notably, the correlation between self-directed learning ability and core competence was the strongest, suggesting that higher self-directed learning ability scores were associated with higher core competence scores. This aligns with the findings of Badakhshan et al. (2025) suggesting a statistically significant association between self-directed learning and clinical competence in nursing students. This association is further supported by a multi-country study conducted across six European countries, which found that graduating nursing students with higher self-directed learning abilities also reported higher self-assessed levels of competence (Visiers-Jiménez et al., 2022). The study, involving 1,746 nursing students from the Czech Republic, Finland, Italy, Portugal, Slovakia, and Spain, also revealed significant cross-country variations, underscoring the importance of considering contextual factors in SDL–competence relationships (Visiers-Jiménez et al., 2022). Theoretically, according to Knowles' theory (Knowles, 1975) of self-directed learning, individuals with a high propensity for self-directed learning are more adept at proactively identifying learning needs, setting goals, and seeking resources—a process that is essential for building core competence. Therefore, self-directed learning ability is not only a significant correlate of core competence but also a variable that shows a strong statistical association with it. This suggests that self-directed learning ability may be a relevant target for educational strategies aimed at supporting core competence in undergraduate nursing education. Furthermore, the significant positive correlation between professional self-concept and core competence indicates that the more positive the students' professional identity, self-esteem, and behavioral orientation, the better their overall performance in clinical practice. Additionally, a moderate positive correlation (*r* = 0.612) was observed between self-directed learning ability and professional self-concept, suggesting that these two constructs are positively interrelated, which may reflect a mutually reinforcing pattern between professional identity and competence.

### 4.3 The mediating role of professional self-concept

Structural equation modeling revealed that professional self-concept partially mediates the relationship between self-directed learning ability and core competence. This indicates that the self-directed learning ability is associated with core competence both directly and indirectly through professional self-concept as a statistical mediator. Similarly, Wang et al. (2025) surveyed 177 nursing interns in a Grade A tertiary hospital in Beijing and found that during clinical placements, nursing students with strong self-directed learning abilities were more adept at identifying their own deficiencies in clinical decision-making, which they subsequently improved by further enhancing their self-directed learning. This closely aligns with the findings of the present study. Specifically, students with high self-directed learning capacities are more inclined to actively explore professional knowledge and reflect on their learning processes, and this pattern is associated with stronger professional identity and beliefs in nursing. This positive professional self-concept, in turn, is associated with stronger comprehensive abilities in clinical practice, reflecting a positive cycle of “Learning Motivation-Professional Identity-Competence Improvement.” A similar logic was observed in a South Korean mixed-methods study, which found that a self-directed clinical practicum enhanced nursing students' self-confidence and satisfaction, with participants reporting both “perceived development of nursing competency” and “establishing nursing identity as a student (Park and Cho, 2022).”

This finding is consistent with Bandura's triadic reciprocal determinism theory, which provides a theoretical framework for understanding the interrelationships among autonomous learning behavior, professional self-concept, and core competence. Based on these findings, and bearing in mind that the present study is cross-sectional and correlational in nature, several educational implications may be considered. First, teaching methods such as project-based learning and flipped classrooms could be integrated into curriculum design to cultivate students' self-directed learning abilities. For example, project-based learning could be operationalized through capstone projects that require students to collaboratively design evidence-based nursing care plans; flipped classroom strategies could be applied to core theoretical courses to stimulate pre-class self-study. Second, professional identity could be supported through professional narrative education and role modeling—for instance, narrative education could be embedded in professional development courses where students write reflective essays about their evolving professional identity. Third, clinical adaptability training could be implemented prior to internships to help students consolidate their professional self-concept in real-world contexts, facilitating a smooth transition from theory to practice. These strategies warrant further investigation in future intervention studies. However, these recommendations are derived from a single-institution convenience sample, and students at this medical university may have unique curricular experiences and cultural backgrounds that influence their self-directed learning and professional identity development. Therefore, the findings and implications should be interpreted with caution when applied to substantially different educational contexts.

In addition to the generalizability concerns noted above, several alternative explanations for the observed associations should also be considered. First, students who perceive themselves as more competent may retrospectively evaluate their learning ability and professional identity more positively, suggesting a potential reverse-direction effect. Second, a supportive educational environment—including curriculum design, faculty mentorship, and peer support—may simultaneously influence all three constructs, making it a potential third-variable confounder. Third, since all measures were self-reported at a single time point, shared method variance may have inflated the observed correlations to some degree. Although Harman's single-factor test did not indicate severe common method bias, this does not entirely rule it out. Future studies employing longitudinal or experimental designs are needed to disentangle these alternative explanations and establish temporal precedence.

## 5 Limitations and future directions

This study has several limitations. First, the cross-sectional design limits the ability to determine causal relationships among the variables. This also limits the generalizability of the research findings to other institutions, regions and nursing education systems. Future studies could employ longitudinal or interventional designs to further verify the mediation pathways. Second, the sample was drawn from a single medical university, which may limit the generalizability of the findings to other institutions, regions, and nursing education systems. Future research should expand the sampling scope to include nursing institutions from diverse regions and educational tiers. Third, this study primarily focused on individual psychological factors. Future research could integrate contextual variables—such as curriculum design, teacher support, and the clinical learning environment—to construct a moderated mediation model, which may contribute to a more comprehensive understanding of nursing students' core competence. Fourth, all data were collected via self-administered questionnaires from the same participants at a single time point. Although Harman's single-factor test was conducted as a preliminary diagnostic, residual common method bias cannot be completely excluded given the cross-sectional self-report design. Future research could incorporate objective clinical performance indicators or multi-informant designs to further reduce this methodological limitation. Fifth, although the final sample of 237 participants meets the minimum recommended sample size for SEM, it remains relatively modest for a mediated model with multiple observed indicators and *post-hoc* modifications. A larger sample would provide more stable parameter estimates and greater statistical power. Future studies should therefore replicate our findings with larger, preferably multi-center, samples to enhance the robustness and generalizability of the model.

## 6 Conclusion

In conclusion, this study provides evidence that nursing undergraduates' self-directed learning ability, professional self-concept, and core competence are at moderate levels and closely interrelated. Furthermore, professional self-concept serves as a partial mediator between self-directed learning ability and core competence. These findings highlight the relevance of professional self-concept in relation to nursing students' core competence, offering practical insights for nursing education and training.

## Data Availability

Due to ethical restrictions approved by the Ethics Committee of the Second Affiliated Hospital of Inner Mongolia Medical University, the raw data are not publicly available. However, anonymized data can be requested from the corresponding author (Yuantong Zang, ysazyt@126.com) upon reasonable request and with approval from the ethics committee.
